# Comprehensive Analysis of the Function, Immune Profiles, and Clinical Implication of m1A Regulators in Lung Adenocarcinoma

**DOI:** 10.3389/fonc.2022.882292

**Published:** 2022-05-30

**Authors:** Guangyao Bao, Tian Li, Xiaojiao Guan, Yao Yao, Jie Liang, Yifang Xiang, Xinwen Zhong

**Affiliations:** ^1^ Department of Thoracic Surgery, First Affiliated Hospital, China Medical University, Shenyang, China; ^2^ School of Basic Medicine, Fourth Military Medical University, Xi’an, China; ^3^ Department of Pathology, Shengjing Hospital, China Medical University, Shenyang, China

**Keywords:** m1A, lung adenocarcinoma, prognosis, immune microenvironment, immunotherapy

## Abstract

**Background:**

Previous studies have demonstrated that transcriptional RNA methyladenosine modification significantly affects tumor initiation and progression. However, clinical implications of N1-methyladenosine (m1A) regulators and their effect on tumor immunity in lung adenocarcinoma (LUAD) are still poorly elucidated.

**Methods:**

Herein, the characteristics of somatic mutation, copy number variation (CNV), DNA methylation, and expression levels of m1A regulators were thoroughly analyzed. We classified 955 lung adenocarcinoma patients into different m1A modification patterns based on an unsupervised consensus clustering algorithm. We then calculated the differences in gene expression, prognosis outcomes, and immune profiles among different m1A clusters. Subsequently, we screened differently expressed genes (DEGs) related to prognosis among different m1A clusters. We identified m1A related gene clusters according to the prognosis-related different expressed genes. We further constructed a scoring standard named the m1A score and comprehensively analyzed the survival outcomes, clinical-pathological features, immune microenvironment, treatment responses of immunotherapy, and drug susceptibility in different m1A score groups.

**Results:**

In total, three different m1A modification patterns were identified, which contained cluster A, B, and C. Among them, cluster A processed the poorest clinical outcomes, the lowest immune cell infiltration rate, and the highest tumor purity score. Then, three m1A gene clusters (gene cluster A, B, C) were speculated. Subsequently, we combined m1A modification patterns and m1A gene cluster to classify lung adenocarcinoma patients into high and low m1A score groups. The low m1A score group was accompanied by higher mortality, higher tumor mutation burden (TMB) and genome mutation frequency, and lower programmed cell death-Ligand 1 (PD-L1) expression and tumor immune dysfunction and exclusion (TIDE) expression. Moreover, the m1A score exhibited positive correlation with almost all immune cells. Finally, common chemotherapeutic and targeted therapy agents exhibited obvious differences in drug susceptibility in different m1A score groups.

**Conclusions:**

Collectively, we explored the potential value of m1A regulators in the prognosis and treatment of lung adenocarcinoma in multiple dimensions and provided some preliminary basis for the follow-up study of m1A regulators in lung adenocarcinoma.

## Introduction

As the cancer with the highest incidence, lung cancer also causes the most cancer-related deaths (1). According to reports, 85% of the total number of new lung cancer each year is non-small cell lung cancer (NSCLC) (2). Currently, lung adenocarcinoma (LUAD), the major type of NSCLC, shows an increasing incidence in young women and non-smokers (3). LUAD is often accompanied by the characteristics of not obvious early symptoms, and prone to hematogenous metastasis and local infiltration. Moreover, patients with advanced lung adenocarcinoma are often accompanied by poor long-term prognosis. In addition, chemotherapy, targeted therapy, and immunotherapy are facing challenges in treatment effectiveness due to the high drug resistance of LUAD (4–6). Therefore, the discovery of molecular markers for early diagnosis and therapeutic efficacy targets of lung adenocarcinoma is an effective way to improve the survival rate of LUAD.

RNA chemical modifications play crucial roles in regulating important cellular processes at the RNA level, including cell differentiation, key cellular signaling pathways, and cell metabolism (7–9). RNA methylation is the major component of RNA chemical modification, which contains N1-methyladenosine (m1A), N3-methylcytosine (m3C), 5-methylcytosine (m5C), and N6-methyladenosine (m6A) (10–12). Among them, N1-methyladenosine (m1A) has been demonstrated to be involved in stabilizing RNA structural, splicing, cell proliferation, and cell aptosis (13, 14). Common m1A regulators contain “writers” (TRMT10C, TRMT61B, and TRMT6/61A), “readers” (YTHDF1, YTHDF2, YTHDF3, and YTHDC1), and “erasers” (ALKBH1 and ALKBH3), which play an essential role in the m1A methylation process (15–17). In general, the “writer” and “eraser” are involved in regulating the state of m1A, while the “reader” acts as m1A binding proteins to access m1A modification information and further identify and combine with methylation sites. The “writer” acts as a methyltransferase complex. Growing evidence indicates that dysregulation of genomic mutation of m1A regulators can influence the process of transcription and translation, resulting in aberrant cell proliferation and tumor initiation (18–21). Moreover, downregulation of ALKBH3 promoted m1A levels and weakened RNA translation levels associated with the accumulation of methylated RNA in the PANC-1 cell line (22). ALKBH3 and ALKBH1 were upregulated in HNSCC and resulted in tumor development (23). However, studies on m1A regulators in LUAD are lacking. Therefore, a multi-dimensional comprehensive assessment of m1A methylation regulators will enhance our understanding of tumorigenesis and the immune microenvironment in LUAD.

In this research, we first investigated the differences in somatic mutation, CNV, DNA methylation, and expression levels of m1A regulators. Further analysis identified three m1A modification patterns and accessed the correlation with tumor microenvironment (TME). Subsequently, the m1A score was developed and used to qualify the m1A modification pattern of a single LUAD patient. Finally, we comprehensively evaluated the prognosis and treatment efficacy of LUAD based on the m1A score system.

## Material and Methods

### Data Collection and Analysis

Nine previously published m1A regulators were included in our research (16, 24–28). Somatic mutation data of LUAD were enrolled from the Cancer Genome Atlas (TCGA) database (https://portal.gdc.cancer.gov/) and visualized by utilizing maftool R package. Subsequently, sequencing data of CNV and DNA methylation were extracted from the Xena database (https://xenabrowser.net/). Transcriptome data and corresponding clinicopathologic characteristics of LUAD were retrospectively curated from TCGA database. Then, three datasets (GSE72094, GSE37745, GSE50081) with clinical information of Gene Expression Omnibus (GEO) were enrolled using GEOquery R package (29), among which GSE37745 and GSE50081 were all RNA sequencing data from the Affymetrix Human Genome U133 Plus 2.0 Array platform. Therefore, we integrated two datasets as a meta-cohort for an independent validation dataset using sva R package for removal of batch effects (30), which contained 235 LUAD samples. Next, RNA expression data of TPM format of the TCGA database and GSE72094 were also combined as a training dataset with sva R package (30), which contained 955 LUAD and 59 normal samples. Furthermore, our study included the anti-PD-L1 treatment cohort IMvigor 210, which contained gene transcriptomic data and clinical information of advanced cancer patients followed by anti-PD-L1 antibody treatment, to further assess the association between m1A modulators and tumor immunity therapy (31).

### Unsupervised Consensus Clustering of Nine m1A Regulators in LUAD

Nine m1A regulators were collected to construct m1A modification patterns, including TRMT6, TRMT61A, TRMT10C, YTHDF1, YTHDF2, YTHDF3, YTHDC1, ALKBH1, and ALKBH3. Unsupervised consensus clustering was performed to identify specific m1A modification patterns. According to gene expression levels of m1A regulators, ConsensusClusterPlus R package was enrolled to clustered 955 LUAD patients into subgroups (32). We set the following clustering parameters: number of cycles = 1000; pItem = 0.8; pFeature = 0.8, and k-means was selected as the clustering algorithm. The clusters that expected the most significant difference in survival were taken into consideration.

### Identification of Immune Cell Infiltration Among Different m1A Modification Patterns

Immune cell infiltration abundance of different m1A cluster groups was identified by a single-sample gene-set enrichment analysis (ssGSEA) algorithm of the GSVA R package (33). Subsequently, enriched pathways for each cluster were also determined.

### Analysis of Differentially Expressed Genes (DEGs) in m1A Cluster Groups

There were 955 lung adenocarcinoma patients classified into three clusters. Then, limma R package was enrolled to identify DEGs in three m1A regulator clusters and adjust p value <0.05 was considered as DEGs.

### Construction of m1A-Related Gene Signatures

Univariate Cox regression analysis of overlapped DEGs among the three m1A regulator clusters was performed to select prognosis-related genes for further analysis using survival R package, with p <0.05 as the threshold. Next, based on prognostic-related genes, an unsupervised consensus clustering algorithm was conducted to classify LUAD patients into different m1A gene clusters. Finally, we performed principal component analysis based on prognosis-related gene expression profiles and identified principal components 1 and 2 as the characteristic scores of each patient. This method mainly includes the scores of gene modules with the most significant positive or negative correlations. In view of this, we established the m1A gene signatures of patients with LUAD based on this formula from previous research: m1Ascore = ∑(PC1i + PC2i), where i represented expression level of prognosis-related gene in different m1A gene clusters.

### Estimation of Drug Sensitivity

Half-maximal inhibitory concentration (IC50) of paclitaxel, gefitinib, vinblastine, and erlotinib were quantified with the pRRophetic R package by ridge regression analysis (34, 35). IC50 indicated the response to the above-mentioned chemotherapy drugs in the TCGA cohort.

### Statistical Analysis

Spearman’s correlation analysis was conducted to estimate composition differences. Wilcoxon signed rank test was applied for comparisons between the two groups. Kaplan-Meier survival curve was implemented for evaluating the survival differences between groups. Statistical analysis was achieved utilizing R software (version 4.02). P <0.05 was taken into consideration statistically.

## Results

### Multi-Omic Landscapes of m1A Regulators in LUAD

**Figure 1:**
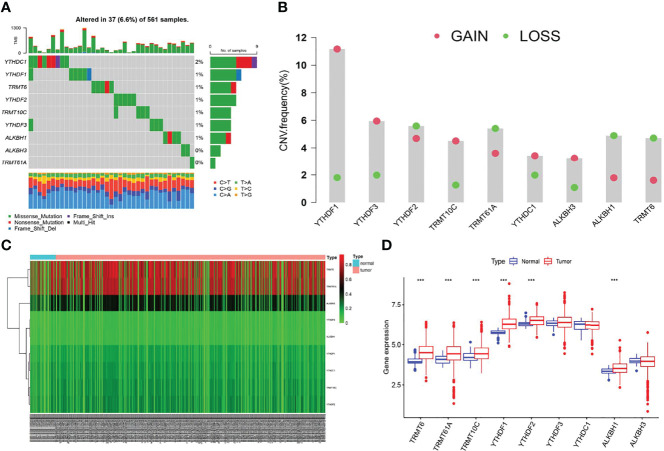
The multi-omic landscapes of nine m1A regulators in LUAD. **(A)** Somatic mutations of nine m1A regulators in TCGA-LUAD. **(B)** The CNV features of m1A regulators in TCGA-LUAD. **(C)** DNA methylation levels of nine m1A regulators in TCGA-LUAD and normal patients (Tumor: 563; Normal: 53). **(D)** Gene expression levels of nine m1A regulators in TCGA-LUAD and normal patients (***P < 0.001).

We first screened the mutation frequency of nine m1A regulators in LUAD. Our results showed that 37 of 561 LUAD samples (6.6%) contained m1A regulators-related mutation, which ranged from 0 to 2% (**Figure 1A**). Further analysis revealed that CNV events occurred frequently in nine m1A regulators. YTHDF1, YTHDF3, TRMT10C, YTHDC1, and ALKBH3 all displayed widespread copy number amplification. Conversely, TRMT6, TRMT61A, YTHDF2, and ALKBH1 exhibited prevalent copy number deletion (**Figure 1B**). Then, the CNV alternation positions of m1A regulators in human chromosome were visualized (**Supplementary Figure S1A**). The differences in the DNA methylation levels of nine m1A regulators in LUAD were subsequently revealed (**Figure 1C**). The results showed TRMT61A, TRMT10C, YTHDF1, YTHDF3, and ALKBH3 were accompanied with higher DNA methylation levels in LUAD (**Supplementary Figure S1B**). Furthermore, the expression levels of YTHDF1, YTHDF2 TRMT6, TRMT61A, TRMT10C, and ALKBH1 were significantly different compared to normal patients (**Figure 1D**). Finally, a comprehensive survival analysis of nine m1A regulators was listed (**Supplementary Figures S1C–F**).

### Identification of Specific m1A Modification Patterns

**Figure 2:**
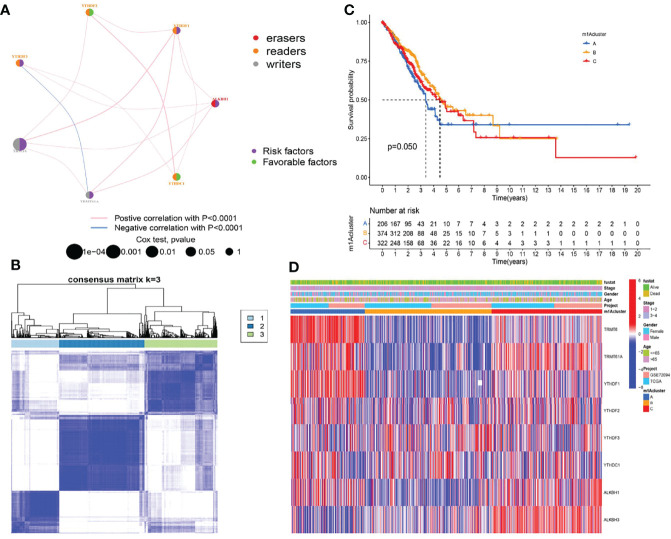
Establishment of three m1A modification patterns. **(A)** The correlation of nine m1A regulators in LUAD. **(B)** Consensus clustering of m1A clusters for LUAD patients in the training cohort. **(C)** Differences in survival outcomes of three m1A clusters in the training cohort. **(D)** Heatmap of three m1A modification patterns in the training cohort.

We first investigated and visualized the interaction network between the nine m1A regulators (**Figure 2A**). A significant interaction network indicated that correlation among different m1A regulators may act as mutually complementary roles in initiation and development of LUAD. According to expression levels of nine m1A regulators, 955 LUAD patients in TCGA and GSE72094 datasets were enrolled in unsupervised clusters for classifying the different m1A modification patterns. We finally determined three different m1A modification patterns: m1Acluster A (222 samples), m1Acluster B (395 samples), and m1Acluster C (338 samples) (**Figure 2B**). With corresponding clinical information, we performed survival analysis among different m1A clusters, and the result showed patients in cluster A exhibited the poorest clinical outcome (**Figure 2C**). Finally, a heatmap systemically depicted the difference in expression levels and clinical pathological features among 3 m1A clusters (**Figure 2D**).

### The Immune Landscape of Different m1A Modification Patterns

**Figure 3:**
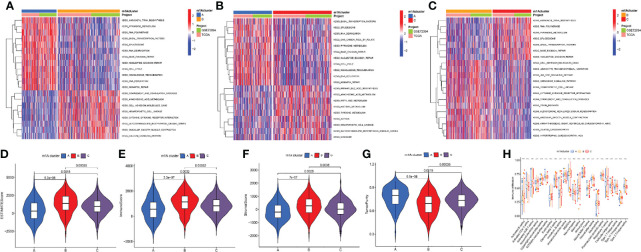
Differences of biological features and immune profiles of three m1A clusters in LUAD. **(A–C)** GSVA results revealed specific biological pathways of three m1A modification patterns. **(A)** Cluster A vs. cluster B; **(B)** cluster A vs. cluster C; **(C)** cluster B vs. cluster C. **(D–G)** Violin plots depicted the distribution of ESTIMATE, immune, and stroma scores as well as tumor purity in three m1A modification patterns. **(H)** Differences in abundance of 23 TME-infiltrating cells under three m1A modification patterns. ** indicates p < 0.01; *** indicates p < 0.001.

The GSVA algorithm was performed to investigate specific biological pathways within the different m1A modification patterns (**Figures 3A–C**). The results of GSVA revealed the m1A cluster A mainly enriched in basal transcription factors, RNA degradation, and cell cycle. The m1A cluster B was strongly associated with complement and coagulation cascades, cell adhesion molecules cams, and cytokine receptor interaction. Moreover, the m1A cluster C exhibited high correlation with cell metabolism, RNA polymerase, and splicesome. Then, we calculated the levels of immune and stromal components across LUAD tissues through the ESTIMATE algorithm. Accordingly, m1A cluster A was accompanied by the highest tumor purity score and the lowest estimate, immune, and stromal score, whereas the m1A cluster B was characterized by the lowest tumor purity score and highest estimate, immune, and stromal score (**Figures 3D–G**). Finally, we systematically qualified the distribution landscape of immune cell infiltration among different m1A modification patterns, with the result indicating that the m1A cluster B displayed the most abundant distribution of adaptive and innate immune cells (**Figure 3H**).

### Investigation of m1A-Related DEGs in LUAD

**Figure 4:**
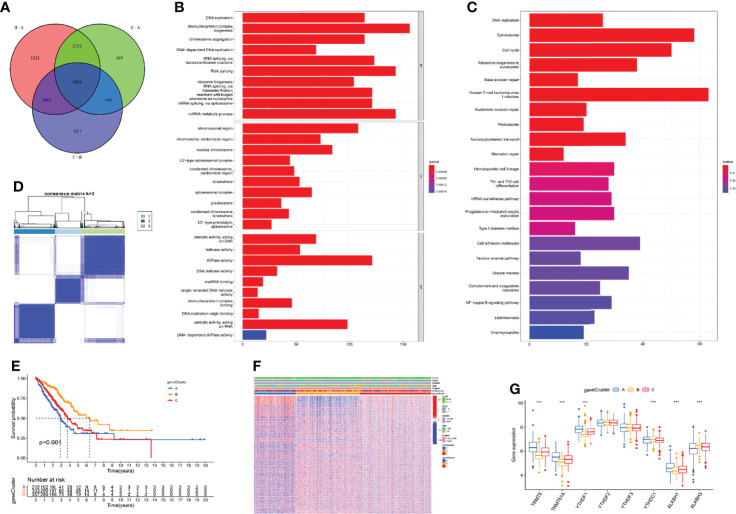
Identification of three m1A gene clusters. **(A)** 2986 DEGs of three m1A modification patterns were shown by Venn diagram. **(B, C)** GO and KEGG results revealed the potential function of 1787 prognosis-related DEGs. **(D)** Consensus clustering of m1A gene clusters for LUAD patients in the training cohort. **(E)** Survival outcome prediction of three m1A gene clusters in the training cohort. **(F)** Heatmap of consensus clustering of 1787 prognosis-related DEGs. **(G)** The different expression levels of m1A regulators in three m1A gene clusters (***P < 0.001).

The above research fully clarified the effects of different m1A modification patterns on the immune microenvironment and clinical outcomes of LUAD patients. To further investigate the underlying impact of m1A regulators in LUAD, we first performed principal component analysis (PCA) based on m1A gene expression and clustering data and revealed that the m1A modification patterns could well reflect the heterogeneity of LUAD patients (**Supplementary Figure S2**). Subsequently, DEGs that intersected between the three m1A clusters were screened and 2986 DEGs were finally uncovered (**Figure 4A**). Then, 1787 prognosis-related DEGs were uncovered by utilizing univariate Cox regression analysis. Gene ontology (GO) enrichment analysis suggested that prognosis-related DEGs were mainly enriched in DNA replication, chromosome segregation, and ATPase activity (**Figure 4B**). Kyoto Encyclopedia of Genes and Genomes (KEGG) enrichment analysis uncovered that prognosis-related DEGs exhibited strong association with DNA replication, cell cycle, and cell adhesion molecules (**Figure 4C**). These results uncovered that m1A-related genes participated in vital cellular pathways and predicted poor clinical outcomes, which may lead to the occurrence and progression of LUAD. Next, we performed unsupervised consensus clusters based on the expression profiling data of prognosis-related DEGs. Then, three m1A-related gene clusters were identified (gene cluster A, B, C) (**Figure 4D**). Subsequently, we found that the LUAD patients divided into m1A gene cluster A were highly correlated with worse survival outcomes (**Figure 4E**). Then, the heatmap comprehensively depicted the clinicopathological characteristics and differences of these subgroups (**Figure 4F**). Finally, we screened the differential expression of m1A regulators among m1A gene clusters. The results revealed that TRMT6, TRMT61A, YTHDF1, and ALKBH1 were significantly upregulated in m1A gene cluster A, whereas YTHDC1 and ALKBH3 were upregulated in m1A gene cluster B (**Figure 4G**).

### Construction of m1A-Related Gene Signatures

**Figure 5:**
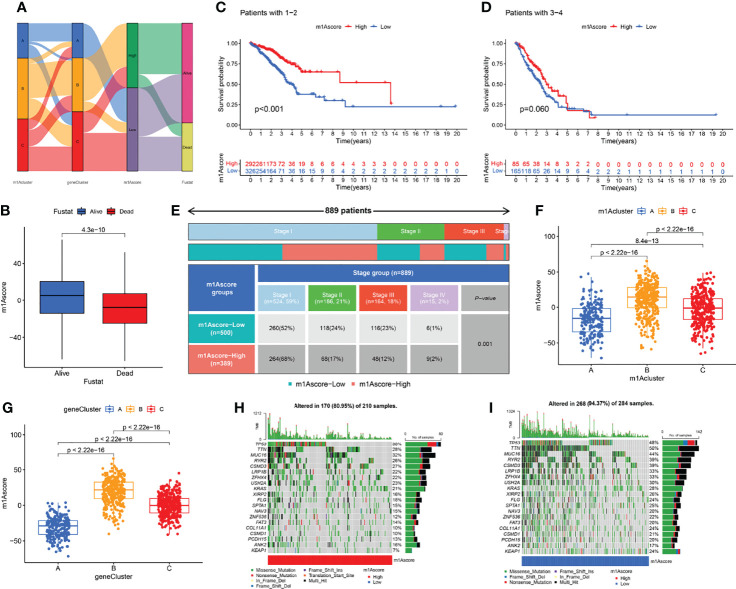
Clinical features in different m1A score groups. **(A)** Alluvial plots depicted the correspondence in m1A clusters, m1A gene clusters, m1A score, and the survival status of patients in LUAD. **(B)** The differences of fustat in different m1A score groups. **(C–E)** Kaplan-Meier curves uncovered differences in m1A scores among LUAD patients at different pathological stages. **(F, G)** The differences of m1A score among three m1A modification patterns and three m1A gen clusters. **(H, I)** The differences of somatic mutation frequency in tumor genomes based on the grouping of m1A scores.

**Figure 6:**
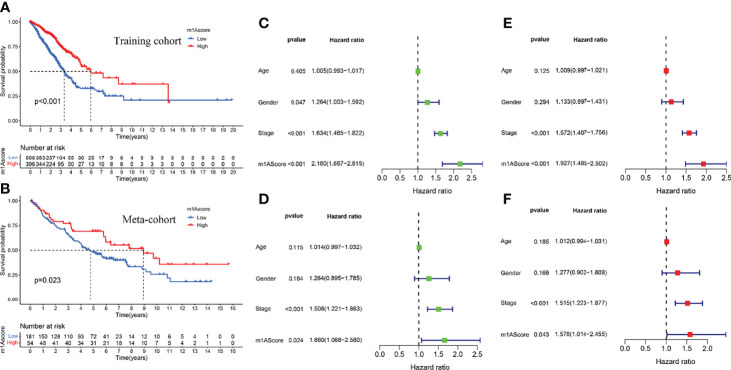
Construction of m1A score and validation. **(A)** Survival outcomes prediction of the m1A score in the training cohort. **(B)** Survival outcomes prediction of the m1A score in the meta-cohort. **(C, E)** Univariate and multivariate analyses revealed the prognostic value of m1A score in the training cohort. **(D, F)** Univariate and multivariate analyses revealed the prognostic value of m1A score in the meta cohort.

The above studies were based on the different m1A classifications of LUAD patients, so it is far from accurate to qualify the impact of m1A modification patterns on specific patient samples. Therefore, we scored LUAD patients according to the m1A modification patterns and gene clusters, named m1A score. We then classified LUAD patients into high and low m1A score groups based on the median m1A score (median m1A score = 4.88). Alluvial plots were performed to depict the correspondence in m1A clusters, m1A gene clusters, m1A score, and the survival status of patients (**Figure 5A**). Then, we noticed that LUAD patients who were surviving showed a higher m1A score than dead LUAD patients (**Figure 5B**). We also uncovered that low m1A score in LUAD patients who were diagnosed with pathological stage I and II often indicated poor prognostic outcomes, whereas the effects of m1A score on predicting prognostic outcomes in LUAD patients with stage III and stage IV showed no statistical difference (**Figures 5C, D**). In addition, based on the information of 889 LUAD patients with clinicopathological stage, we found significant differences in m1A scores among LUAD patients with different pathological stages (**Figure 5E**). Moreover, m1A scores in different m1A modification patterns and gene clusters also showed significant differences (**Figures 5F, G**). Next, we comprehensively screened the differences in somatic mutations in tumor genomes based on the grouping of m1A scores, which indicated that the low m1A score group exhibited the higher mutation frequency (**Figures 5H, I**). Further, we identified that low m1A score subpopulations were often accompanied by high mortality, which was consistent with the result of the meta-cohort (**Figures 6A, B**). Subsequently, univariate and multivariate analysis uncovered m1A scores possessed the potential in independently predicting LUAD prognosis (**Figures 6C, E**), which was also validated in the meta-cohort (**Figures 6D, F**).

### Identifying and Comparing the Immune Profiles of Different m1A Score Groups

**Figure 7:**
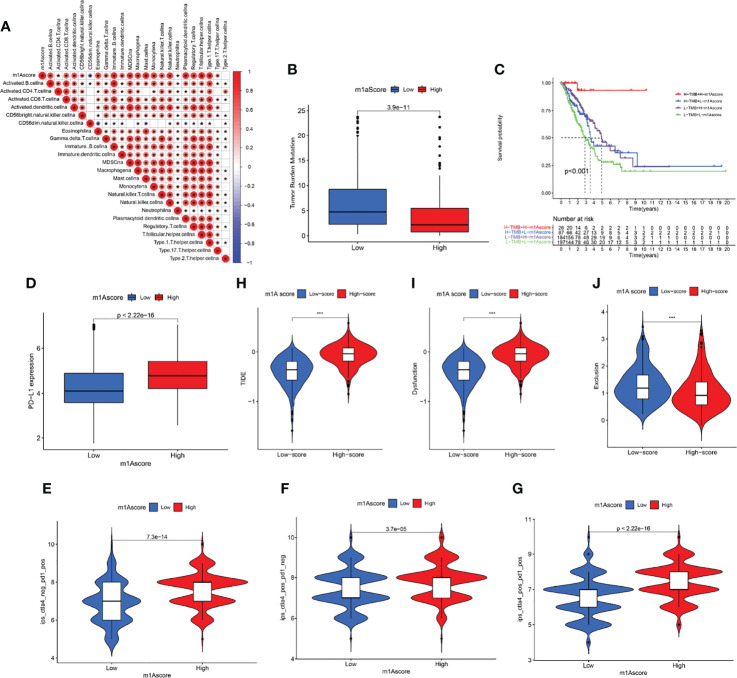
Profile of differences in the immune microenvironment in different m1A score groups. **(A)** Correlation between m1Ascore and immune-related cellular components. Blue indicates negative correlation; red indicates positive correlation; * indicates P < 0.05. **(B)** Comparisons of TMB score in different m1A score groups. **(C)** Overall survival analysis of different m1A score and TMB score groups. **(D)** PD-L1 expression levels in different m1A score groups. **(E–G)** Treatment effects of CTLA-4 or PD-1 and combined CTLA-4 and PD-1 were evaluated in patients with high and low m1A scores. **(H–J)** TIDE, dysfunction, and exclusion scores in different m1A score groups.

We first determined the association of m1A score with immune cells. The result uncovered that m1A score was positively correlated with almost all immune cells except CD56 natural killer cell (**Figure 7A**). These findings revealed that m1Ascore could be used to effectively evaluate m1A modification patterns and the differences in immune cell infiltration in a single LUAD patient. We also investigated the relationship between m1A score and tumor burden mutation (TMB) and noticed that the low m1A score group exhibited high-level of TMB (**Figure 7B**). Moreover, the m1A score was negatively correlated with TMB (**Supplementary Figure S3**). Further analysis uncovered that LUAD patients in low m1A score and low TMB group displayed the worst survival outcomes (**Figure 7C**). Next, the interaction of m1A score and PD-L1 expression was investigated. Patients with LUAD in the low m1A score group were accompanied by low levels of PD-L1 expression (**Figure 7D**). We comprehensively evaluated the immunotherapy response of patients with LUAD based on the m1A score. High m1A score group patients expressed therapeutic advantage to CTLA-4 and PD-1 monotherapy (**Figures 7E, F**). Similarly, LUAD patients with high m1A score showed treatment advantages to CTLA-4 and PD-1 combined treatment (**Figure 7G**). In addition, we determined immunotherapy efficacy in patients with different m1A score groups based on the TIDE database. The results indicated that the TIDE expression showed significant difference in the high and low m1A score groups (**Figure 7H**). Further, we noticed that LUAD patients in high m1A score group were accompanied by higher tumor dysfunction score and lower tumor exclusion score (**Figures 7I, J**). Finally, we speculated the ability of the m1A score in predicting patients’ immune effects based on the IMvigor210 immunotherapy cohort. Interestingly, we noticed that the immunotherapy efficacy in different m1A score groups exhibited no statistical difference (**Supplementary Figure S4**).

### Drug Susceptibility Prediction in Different m1A Score Groups

**Figure 8:**
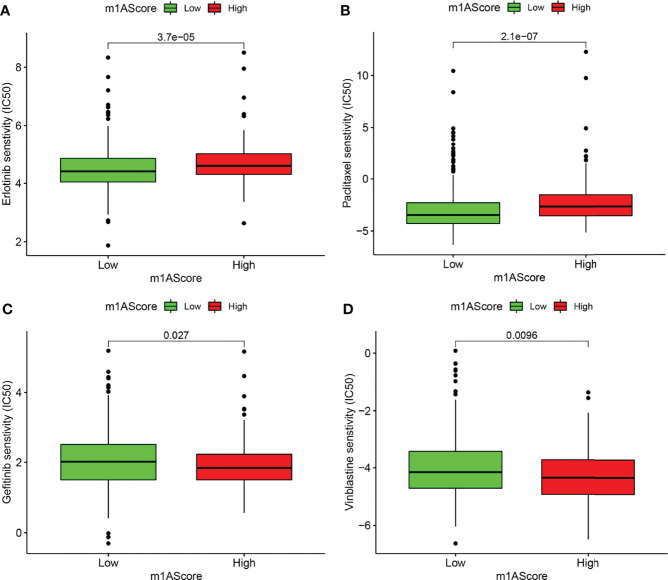
Drug susceptibility prediction in different m1A score groups. **(A–D)** Comparisons of sensitivity to erlotinib, paclitaxel, gefitinib, and vinblastine in different m1A score groups.

Chemotherapy and targeted therapy were gradually applied in treatments for patients with advanced LUAD. It is of great significance to evaluate the responses of certain drugs in different subpopulations. Herein, we identified the treatment responses of some drugs that were widely used in the treatment of LUAD. As shown in **Figure 8**, the high m1A score group possessed prominently high IC50 values of erlotinib and paclitaxel, indicating that this subpopulation showed higher sensitivity to these therapeutic agents, whereas patients in the low m1A score group showed therapeutic superiority to gefitinib and vinblastine. The above research results provided more reference values for formulating personalized treatment strategies for LUAD patients.

## Discussion

Previous studies have confirmed that m1A methylation modification significantly affects the occurrence and development of tumors (36, 37). However, there are few studies exploring the role of m1A modification in the tumorigenesis of LUAD. Herein, we first revealed the underlying role of m1A modification in LUAD from multiple perspectives. Then, we identified the differences of TME cells infiltration among three m1A modification patterns. Subsequently, the m1A score system was constructed and used to qualify the m1A modification pattern of a single LUAD patient. Finally, we comprehensively evaluated the prognosis and treatment efficacy of LUAD based on the m1A score system.

In this study, the characteristics of somatic mutation, copy number variation (CNV), DNA methylation, and gene expression levels of nine m1A regulators in TCGA-LUAD cohort were screened. We found that 37 of 561 LUAD samples (6.6%) contained m1A regulators-related mutation, with mutation frequencies ranging from 0 to 2%, and YTHDC1 occupied the top mutation frequency. Previous research suggested that YTHDC1 deficiency could significantly increase the level of alternative splicing defects in mouse oocytes (38). Further, six m1A regulators displayed significantly high expression levels in LUAD patients. Moreover, high expression of ALKBH1, TRMT6, and TRMT61A was found to be indicative of poor clinical outcomes, which were consistent with their high expression in LUAD. Further studies on the CNV signature of nine m1A regulators showed that YTHDF3, TRMT10C, YTHDC1, YTHDF1, and ALKBH3 displayed copy number amplification, while TRMT6, TRMT61A, YTHDF2, and ALKBH1 exhibited copy number deletion. Genomic alternations of the m1A regulators in LUAD could be due to abnormal gene expression, which contributed to tumor development.

Then, three m1A modification patterns were revealed, named m1A clusters A, B, C. Within these modification patterns, cluster A displayed the poorest long-term survival outcomes. Meanwhile, cluster A was accompanied by the lowest estimate, immune, and stromal score, and the highest tumor purity. Further analysis revealed cluster A showed lower infiltration of immune cells. The purity of the tumor depends on the proportion of tumor cells in TME, and its level affects the prognosis of cancer patients (39, 40). Hence, we speculated that the poor long-term prognosis of LUAD patients in m1A cluster A may be related to high tumor purity and suppression of immune function. GSVA analysis revealed the m1A cluster A mainly enriched in basal transcription factors, RNA degradation, and cell cycle, which may be involved in the progression of LUAD (41, 42). In view of this, we believe that different m1A modification patterns may shape the tumor immune microenvironment (TIME) of LUAD, thereby potentially affecting the prognosis of LUAD.

Based on prognostic-related DEGs among different m1A modification patterns, we revealed three m1A gene clusters. Similarly, the m1A gene cluster exhibited the poorest clinical outcome. The m1A scoring system was subsequently constructed to assess the impact of m1A methylation on single LUAD patients. Survival analysis revealed that LUAD patients with low m1A score suggested high mortality. In addition, we found LUAD patients classified into m1A cluster A and m1A gene cluster A groups were accompanied by the lowest m1A score. In view of this, we noticed that LUAD patients showed different clinical prognosis with different grouping methods based on m1A modification patterns, which revealed that m1A had a clear prognostic value for LUAD patients. TMB was characterized as an effective indicator for prediction of clinical response to immunotherapy (43). Our data suggested that low m1A score group showed a high-level of TMB, while LUAD patients with both low m1A score and low TMB exhibited poor clinical prognosis. Moreover, m1A score was positively correlated with almost all immune cells. The above findings suggested m1A score may have the ability to predict prognosis of LUAD and evaluate the tumor immune microenvironment and immune response of LUAD.

Immunotherapy is gradually becoming an important treatment for advanced LUAD. PD-L1 has become a powerful biomarker to assess the efficacy of immune checkpoint inhibitor (ICI) in LUAD patients recently (44). High expression of PD-L1 often predicts better treatment response to ICIs (6, 44, 45). Here, we noticed that high m1A score group exhibited high expression level of PD-L1, which uncovered LUAD patients with high m1A score may occupy a higher priority for anti-PD-L1 therapy. The above results indicated that predicting anti-PD-L1 efficacy based on m1A score required more clinical trials to verify. Moreover, we found that high m1A score patients presented higher sensitivity to erlotinib and paclitaxel, and patients with low m1A score had higher priority to gefitinib and vinblastine, providing a reference for the choice of the optimal chemotherapeutic or targeted therapeutic regimen.

## Conclusion

To sum up, our study characterizes m1A regulators in LUAD from multiple dimensions and qualified its significant role in predicting prognosis value and immune performance. Further analysis revealed the interaction between m1A score and immune microenvironment. Importantly, we provided some preliminary basis for the follow-up study of m1A regulators in lung adenocarcinoma. Nevertheless, their potential significance as prognostic indicators and therapeutic guidance value of LUAD is worthy of further study.

## Data Availability Statement

Publicly available datasets were analyzed in this study. This data can be found here: TCGA-LUAD, GSE72094, GSE37745, and GSE50081.

## Ethics Statement

The studies involving human participants were reviewed and approved by Ethics committee of China Medical University. The patients/participants provided their written informed consent to participate in this study.

## Author Contributions

GYB performed the statistical analyses and wrote the manuscript. GYB completed all of the data entry and provided assistance for the data analysis. GYB, XJG, YY, JL and XWZ were responsible for the diagnosis and clinical assessment of the participants. XW Z and TL designed and wrote the study protocol and reviewed the manuscript. XJG and YX participated the revision of this manuscript. In addition, YY and JL offered many constructive opinions on this study and provided a critical revision of the manuscript for important intellectual content. All authors contributed to and approved the final manuscript.

## Funding

This work was supported by Wu Jieping Medical Foundation [grant number 320.6750.2020-17-7].

## Conflict of Interest

The authors declare that the research was conducted in the absence of any commercial or financial relationships that could be construed as a potential conflict of interest.

## Publisher’s Note

All claims expressed in this article are solely those of the authors and do not necessarily represent those of their affiliated organizations, or those of the publisher, the editors and the reviewers. Any product that may be evaluated in this article, or claim that may be made by its manufacturer, is not guaranteed or endorsed by the publisher.

## Supplementary Material

Supplementary Figure 1 Comprehensive analysis of nine m1A regulators in lung adenocarcinoma multi-omics. **(A)** The CNV alternation positions in human chromosome. **(B)** The DNA methylation levels in TCGA-LUAD and normal patients (*P < 0.05; **P < 0.01; ***P < 0.001). **(C–F)** Survival outcome prediction of ALKBH1, TRMT6, TRMT61A, and YTHDF2 in the training cohort.

Supplementary Figure 2 Principal component analysis revealed that the m1A modification patterns could well reflect the heterogeneity of LUAD patients.

Supplementary Figure 3 TMB exhibited a significantly negative correlation with m1A score (R = -0.37, p < 2.2e-16).

Supplementary Figure 4 Immune responses of different m1A score groups exhibited no statistical difference in IMvigor210 immunotherapy cohort (p = 0.78).

## Abbreviations

NSCLC, non-small cell lung cancer; LUAD, lung adenocarcinoma; m1A, N1-methyladenosine; TCGA, The Cancer Genome Atlas; GEO, Gene Expression Omnibus; TPM, transcripts per kilobase million; CNV, copy number variation; IC50, half-maximal inhibitory concentration; ssGSEA, single-sample gene-set enrichment analysis; ESTIMATE, Estimation of Stromal and Immune Cells in Malignant Tumors using Expression Data; PCA, principal component analysis; GO, gene ontology; KEGG, Kyoto Encyclopedia of Genes and Genomes; TMB, tumor mutation burden; PD-L1, programmed cell death-Ligand 1; TIDE, tumor immune dysfunction and exclusion; TIME, tumor immune microenvironment; ICI, immune checkpoint inhibitor; FPKM, fragments per kilobase of transcript per million mapped reads.
