# Supplementary figures and images for: Comprehensive Analysis of the Function, Immune Profiles, and Clinical Implication of m1A Regulators in Lung Adenocarcinoma
Source: Front Oncol. 2022 May 30;12:882292. doi: 10.3389/fonc.2022.882292 (PMC9197195; doi:10.3389/fonc.2022.882292)

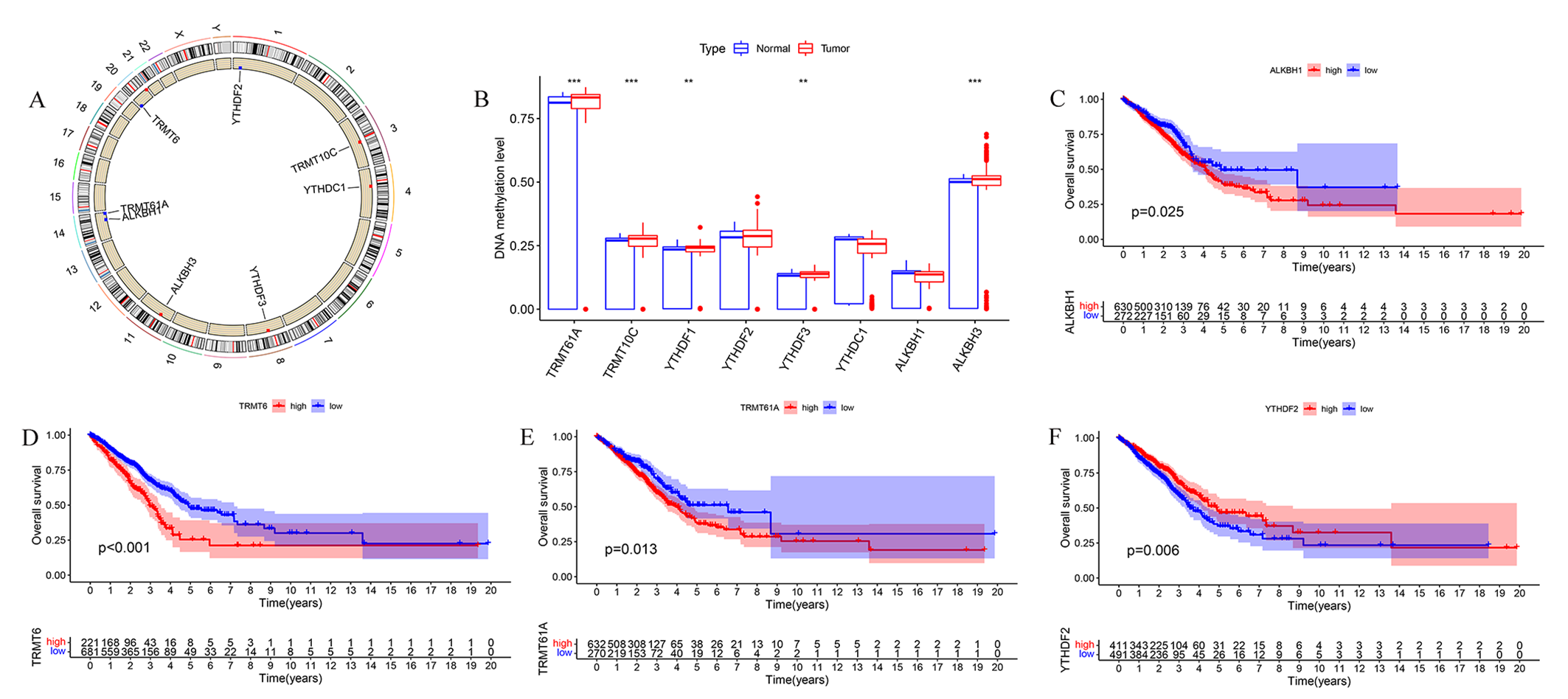

Supplement: Supplementary Figure 1 — Comprehensive analysis of nine m1A regulators in lung adenocarcinoma multi-omics. (A) The CNV alternation positions in human chromosome. (B) The DNA methylation levels in TCGA-LUAD and normal patients (*P < 0.05; **P < 0.01; ***P < 0.001). (C–F) Survival outcome prediction of ALKBH1, TRMT6, TRMT61A, and YTHDF2 in the training cohort. [file Image_1.tif]

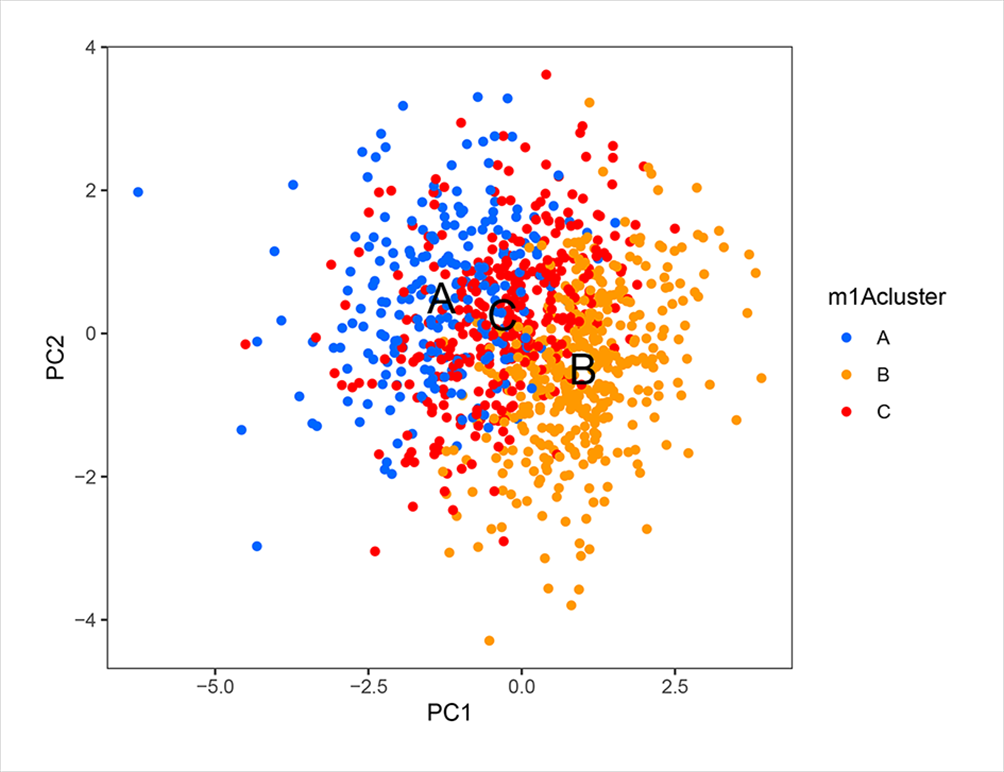

Supplement: Supplementary Figure 2 — Principal component analysis revealed that the m1A modification patterns could well reflect the heterogeneity of LUAD patients. [file Image_2.tif]

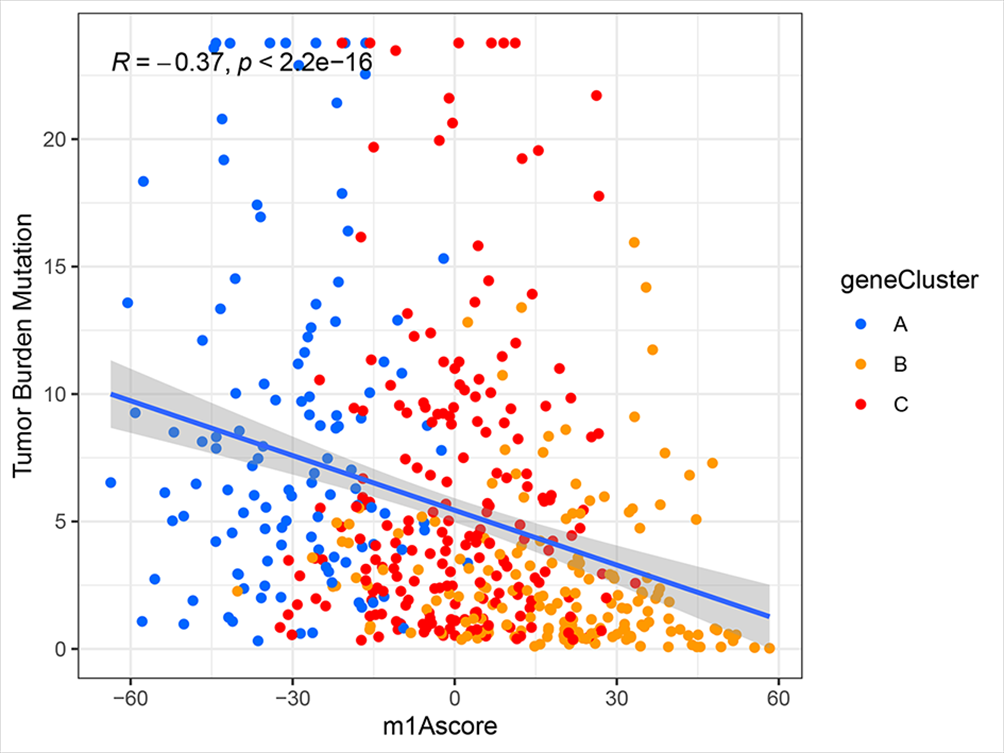

Supplement: Supplementary Figure 3 — TMB exhibited a significantly negative correlation with m1A score (R = -0.37, p < 2.2e-16). [file Image_3.tif]

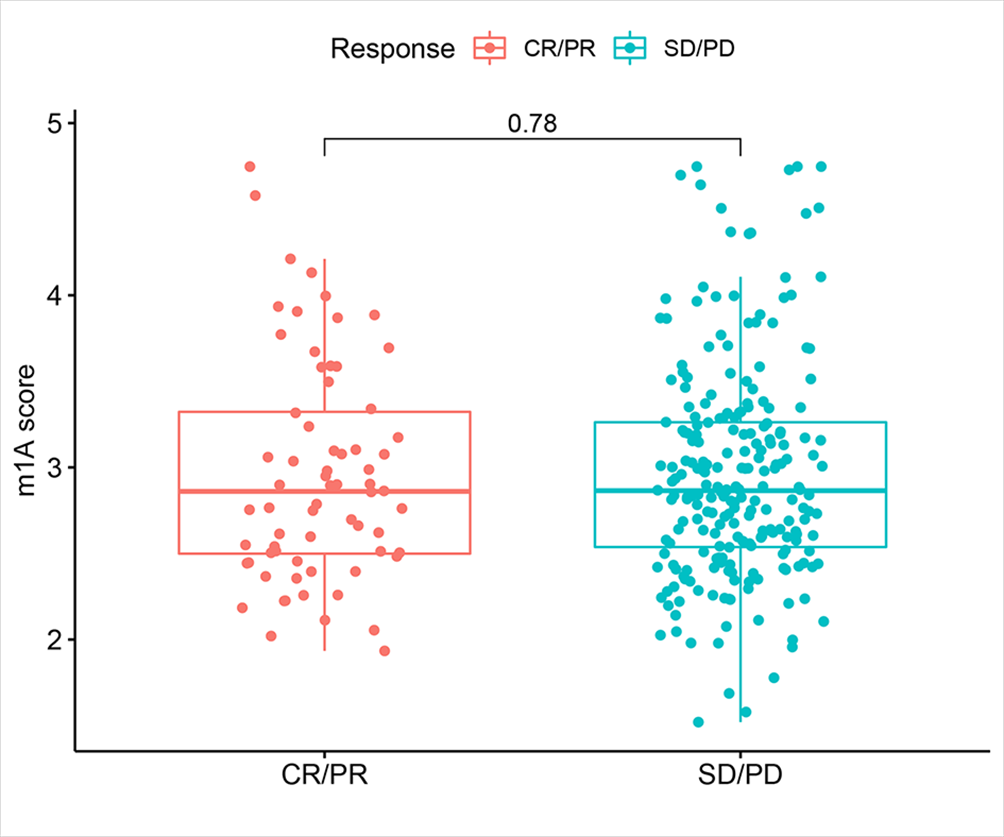

Supplement: Supplementary Figure 4 — Immune responses of different m1A score groups exhibited no statistical difference in IMvigor210 immunotherapy cohort (p = 0.78). [file Image_4.tif]
